# Use of Potential Probiotic Lactic Acid Bacteria (LAB) Biofilms for the Control of *Listeria monocytogenes, Salmonella* Typhimurium, and *Escherichia coli* O157:H7 Biofilms Formation

**DOI:** 10.3389/fmicb.2016.00863

**Published:** 2016-06-10

**Authors:** Natacha C. Gómez, Juan M. P. Ramiro, Beatriz X. V. Quecan, Bernadette D. G. de Melo Franco

**Affiliations:** ^1^Department of Food and Experimental Nutrition, Food Microbiology, Faculty of Pharmaceutical Sciences, Food Research Center, University of São PauloSão Paulo, Brazil; ^2^University of JaénJaén, Spain

**Keywords:** biofilm, probiotic, lactic acid bacteria, exclusion, pathogens, biocontrol

## Abstract

Use of probiotic biofilms can be an alternative approach for reducing the formation of pathogenic biofilms in food industries. The aims of this study were (i) to evaluate the probiotic properties of bacteriocinogenic (*Lactococcus lactis* VB69, *L. lactis* VB94, *Lactobacillus sakei* MBSa1, and *Lactobacillus curvatus* MBSa3) and non-bacteriocinogenic (*L. lactis* 368, *Lactobacillus helveticus* 354, *Lactobacillus casei* 40, and *Weissela viridescens* 113) lactic acid bacteria (LAB) isolated from Brazilian’s foods and (ii) to develop protective biofilms with these strains and test them for exclusion of *Listeria monocytogenes, Escherichia coli* O157:H7, and *Salmonella* Typhimurium. LAB were tested for survival in acid and bile salt conditions, surface properties, biosurfactant production, β-galactosidase and gelatinase activity, antibiotic resistance and presence of virulence genes. Most strains survived exposure to pH 2 and 4% bile salts. The highest percentages of auto-aggregation were obtained after 24 h of incubation. Sixty-seven percentage auto-aggregation value was observed in *W. viridescens* 113 and *Lactobacillus curvatus* MBSa3 exhibited the highest co-aggregation (69% with *Listeria monocytogenes* and 74.6% with *E. coli* O157:H7), while the lowest co-aggregation was exhibited by *W. viridescens* 113 (53.4% with *Listeria monocytogenes* and 38% with *E. coli* O157:H7). Tests for hemolytic activity, bacterial cell adherence with xylene, and drop collapse confirmed the biosurfactant-producing ability of most strains. Only one strain (*L. lactis* 368) produced β-galactosidase. All strains were negative for virulence genes *cob, ccf, cylLL, cylLs, cyllM, cylB, cylA* and *efaAfs* and gelatinase production. The antibiotic susceptibility tests indicated that the MIC for ciprofloxacin, clindamycin, gentamicin, kanamycin, and streptomycin did not exceed the epidemiological cut-off suggested by the European Food Safety Authority. Some strains were resistant to one or more antibiotics and resistance to antibiotics was species and strain dependent. In the protective biofilm assays, strains *L. lactis* 368 (bac-), *Lactobacillus curvatus* MBSa3 (bac+), and *Lactobacillus sakei* MBSa1 (bac+) resulted in more than six log reductions in the pathogens counts when compared to the controls. This effect could not be attributed to bacteriocin production. These results suggest that these potential probiotic strains can be used as alternatives for control of biofilm formation by pathogenic bacteria in the food industry, without conferring a risk to the consumers.

## Introduction

Lactic acid bacteria (LAB) constitute part of the autochthonous microbiota of many types of foods. They are defined as a cluster of lactic-acid-producing, low G + C%, non-spore-forming, Gram-positive rods and cocci and catalase-negative bacteria which share many biochemical, physiological, and genetic properties (Abriouel et al., 2012). This group of bacteria has a particular interest for food industries due to their technological properties, being often used as starter cultures to produce fermented products (Lahtinen et al., 2011). Many reports have shown that traditional fermented foods are rich sources of LAB with probiotic characteristics (Liu et al., 2011; Favaro et al., 2014; Palomino et al., 2015).

According to FAO/WHO (2006), probiotics are live microorganisms which administered in adequate amounts confer a health benefit on the host (FAO/WHO, 2006). The principal functional properties of probiotics include tolerance to acid and bile, adherence to epithelial surfaces, and antagonistic activity toward intestinal pathogens. Probiotics may confer their health benefits by several mechanisms; by contributing to colonization resistance, reinforcing the intestinal barrier (i.e., tight junction expression, secretion of mucus, and antimicrobial peptides), modulating the immune system and instructing the intestinal microbiota composition and activity (Wan et al., 2015). This is based on either direct cell–cell contact, secreting various molecules and/or microbial cross-feeding (Jonkers, 2016). Auto-aggregation of probiotic strains seems to have influence on their adhesion to intestinal epithelial cells, while co-aggregation with pathogens may prevent colonization in the gut and their consumption reduces the viable number of pathogens while strengthening body natural defenses (Savard et al., 2011). Del Re et al. (2000) demonstrated that auto-aggregation is strongly related to adhesion. In addition, adhesion of probiotic bacteria to mucosa is one of the mechanisms by which they can overcome competition with other microorganisms. Nevertheless, production of bacteriocins and other antimicrobial substances by bacteria in biofilms and adhered to mucosal surfaces is considered relevant for the displacement of pathogens, as demonstrated in gastrointestinal tract (GIT) models (Ganzle et al., 1999). Bacteriocin-producing *Lactobacillus curvatus* LTH 1174 provided protection against *E. coli* LTH 1600 and *Listeria innocua* DSM20649 invasion during transit through in a dynamic model of the human stomach and small intestine (GIT model; Ganzle et al., 1999) and bacteriocin-producing *Lactobacillus sakei* 2a protected gnotobiotic mice against experimental challenge with *Listeria monocytogenes* (Bambirra et al., 2007). These data suggest that bacteriocin-producing lactobacilli prevent new strains from invading or maintaining stable populations in the colon. Therefore, bacteriocin production is often considered a probiotic trait in this context.

Studies carried out both in culture media and foods have shown that bacteriocins produced by probiotic or potentially probiotic LAB can act synergistically or have an additive effect in the antimicrobial activity when combined with other antimicrobials (Viedma et al., 2010; Gómez et al., 2012). Interestingly, LAB may simultaneously secrete organic acids, bacteriocins, and biosurfactants (Kanmani et al., 2013). The precise role of these compounds on other bacterial populations present in biofilms is not yet known, but it is well recognized that bacteriocins have stronger antimicrobial activity under acidic conditions (Gálvez et al., 2010).

The presence of biofilms is a relevant risk factor in the food industry due to the potential contamination of food products with pathogenic and spoilage microorganisms. Biofilms can be formed on surfaces becoming permanent reservoirs of bacteria. Most important, biofilms may act as reservoirs of pathogenic and spoilage bacteria, in which these microorganisms can persist against the cleaning and disinfection processes. For example, contamination of equipment with biofilms was a contributing factor to 59% of food-borne disease outbreaks investigated in France (Midelet and Carpentier, 2004). The presence of biofilms is common in food industry and represents a concern because bacteria can adhere to almost any type of surface, such as plastic, metal, glass, soil particles, wood food products (Gandhi and Chikindas, 2007).

*Listeria monocytogenes* is commonly found in food-processing environment, and it has been isolated from both meat and dairy processing plants (Winkelströter et al., 2013) and Mendonça et al. (2012) also demonstrated that *E. coli* O157:H7 has the potential to form biofilm on different surfaces commonly used in food industry. Common sites for the presence of *Salmonella* spp. in food-processing plants are filling or packaging equipments, floor drains, walls, cooling pipes, conveyors, collators for assembling product for packaging, racks for transporting products, hand tools or gloves, freezers, etc, which are usually made of plastics (Pompermayer and Gaylarde, 2000). In addition, a study of 122 *Salmonella* strains indicated that all had the ability to adhere to plastic microwell plates and that; generally, more biofilm was produced in low nutrient conditions, as can be found in specific food-processing environments, compared to high nutrient conditions (Stepanović, 2004).

The increased resistance of biofilm cells to biocides can be partially due of the exopolymeric matrix interference and this can explains why the disinfectant most effective to planktonic cells is not necessarily the most active against biofilm cells (Van Houdt and Michiels, 2010). *Listeria monocytogenes* cells residing in so-called refuge sites such as cracks, worn equipment and in hard to reach places such as complex machinery may be subjected to suboptimal disinfection concentrations allowing them to survive and possibly adapt to cleaning and sanitation treatments (Carpentier and Cerf, 2011).

Recent trends in the transmission and emergence of resistant pathogenic bacteria through the food chain reinforce the need to investigate several alternatives for disinfection. For this reason, there is a great interest in the development of novel strategies using natural products to control the persistence of pathogens associated with surfaces or equipment especially in food industry. Therefore, biofilms formed by LAB present in foods, agricultural products or in the GIT of mammals and used as starters in food manufacturing, may offer a promising means to counteract the establishment of pathogenic biofilms (Winkelströter et al., 2013).

A very promising approach for the control of biofilm formation is the use of probiotics to colonize hard surfaces in order to counteract the proliferation of other bacterial species, based on the competitive exclusion principle (Falagas and Makris, 2009; Hibbing et al., 2010). This concept has been designated as biocontrol when the application is antagonistic toward a certain pathogen (Gatesoupe, 1999). LAB successfully reduced *Listeria monocytogenes* in a ready-to-eat poultry processing plant (Zhao et al., 2013) and lactobacilli with biofilm-forming aptitudes were able to control *Listeria monocytogenes* on abiotic surfaces (Pérez-Ibarreche et al., 2014). In addition, several studies have shown that bacteriocin-producing LAB improved the bactericidal effect of biocides on bacterial biofilms (Lobos et al., 2009; Gómez et al., 2012).

Application of bacteriocins and/or their producer strains for inhibition of biofilm formation and/or killing of cells embedded in biofilms is a novel field of research. The objectives of this study were to evaluate the potential probiotic traits of LAB isolated from different fermented Brazilian products and their inhibition effect against *Escherichia coli* O157:H7, *Listeria monocytogenes*, and *S.* Typhimurium biofilm formation. Tolerance to low pH and bile salts, surface properties (aggregation and co-aggregation), biosurfactant production, gelatinase activity, antibiotic resistance and virulence genes absence were evaluated as probiotic properties of the studied LAB.

## Materials and Methods

### Bacterial Strains and Growth Conditions

The study was conducted with eight LAB strains isolated from foods (**Table 1**): bacteriocin producers *Lactococcus lactis* VB69 and VB94 were isolated from Brazilian charqui (Bíscola et al., 2013) and *Lactobacillus sakei* MBSa1 and *Lactobacillus curvatus* MBSa3 were isolated from salami (Barbosa et al., 2015). Non-bacteriocin producers *Lactococcus lactis* 368, *Lactobacillus helveticus* 354 isolated from goat cheese and *Lactobacillus casei* 40 and *W. viridescens* 113 isolated from ripened cheese (unpublished). The strains were identified by 16S rDNA gene sequencing, according to Cibik et al. (2000), in a CEQ2000 XL DNA Analysis System (Beckman Coulter, Brea, CA, USA). LAB strains were cultivated in De Man et al. (1960) broth (Oxoid, Basingstoke, England) at 30°C for 18 h. *E. coli* O157:H7 ATCC 35150, *Listeria monocytogenes* ATCC 7644 and *S.* Typhimurium ATCC 14028 were cultured in trypticase soy broth (TSB, Oxoid, Basingstoke, England) at 37°C for 20 h. All strains were maintained at -80°C in the appropriate cultivation broth containing 20% (v/v) glycerol.

**Table 1:** Bacterial strains used in this study.

Identification code	Strain	Isolation source	Bacteriocine production
MBSa1	*Lactobacillus sakei*	Salami	Sakacine A
MBSa3	*Lactobacillus curvatus*	Salami (Barbosa et al., 2015)	Sakacine P
VB69	*Lactococcus lactis*	Charque (Bíscola et al., 2013)	Nisin Z
VB94	*Lactococcus lactis*	Charque (unpublished)	Nisin Z
40	*Lactobacillus casei*	Ripened cheese (unpublished)	No producer
352	*Lactobacillus helveticus*	Goat cheese (unpublished)	No producer
368	*Lactococcus lactis*	Goat cheese (unpublished)	No producer
113	*Weisella viridescens*	Ripened cheese (unpublished)	No producer

### Auto-Aggregation and Co-Aggregation Assays

Aggregation abilities of LAB strains were studied as described by Collado et al. (2008), with some modifications. Bacterial cells from an overnight culture were harvested by centrifugation (5,000 × *g*, 20 min, 4°C), washed twice with phosphate-buffered saline PBS pH 7.1 (10 mM Na_2_HPO_4_, 1 mM KH_2_PO_4_, 140 mM NaCl, 3 mM KCl) and suspended in the same buffer. Absorbance (*A*_600__nm_) was adjusted to 0.25 ± 0.05 in order to standardize the number of bacteria (10^7^–10^8^ CFU/ml). The optical density (OD_600__nm_) of a homogenized bacterial suspension was first recorded then repeated on the same suspension left to rest for 24 h at 37°C without vortexing. The aggregation percentage was expressed as [1 - (*A*_Time_/*A*_0_) × 100] where *A*_Time_ represents the absorbance of the mixture at 24 h and *A*_0_, absorbance at time 0.

For the co-aggregation assays, LAB bacterial suspensions prepared as described above were mixed with equal volumes (500 μl) of the cultures of the pathogens listed in Section “Bacterial Strains and Growth Conditions.” Mixtures were incubated at 37°C without agitation, and absorbance (OD_600__nm_) measured after 24 h at 37°C. The percentage of co-aggregation was calculated as [(*A*_pathog_ + *A*_LAB_)/2 - (*A*_mix_)/(*A*_pathog_ + *A*_LAB_)/2] × 100 (Handley et al., 1987), where *A*_pathog_ and *A*_LAB_ represent the absorbance in the tubes containing only the pathogen or the LAB strain, respectively, and *A*_mix_ represents the absorbance of the mixture at 24 h (García-Cayuela et al., 2014).

### Tolerance to Bile Salts and Acidic pH

The LAB strains were tested for bile salt tolerance (0–10%) and survival at low pH (1.5–3) according to Millette et al. (2008). The bile salt tolerance was ascertained in MRS agar containing a commercial preparation of bile salts normally used to inhibit the growth of Gram-positive bacteria in broth (Sigma–Aldrich, B-3426). The bile salt mixture was added in concentrations varying from 0 to 10% with increments of 1%. Another bile salt preparation (LP 0055; Oxoid, Basingstoke, England) was also evaluated in concentrations varying from 0 to 20% with increments of 4% to avoid differences between the different compounds. The MRS agar containing the bile salts was autoclaved for 15 min at 121°C, cooled, and plated. Aliquots of overnight MRS broth cultures (100 μl of bacteria in the stationary phase obtained after 24 h of growth) were inoculated onto the surface of the bile-salt-containing MRS agar, and incubated at 37°C for 72 h. The plates were examined visually for bacterial growth as a lawn, indicating resistance to bile salts in the tested concentration. For determination of acid tolerance, 1 ml overnight MRS broth cultures were inoculated onto 19 ml of simulated gastric fluid (3.2 g/l pepsin and 2 g/l NaCl) adjusted to different pHs (1.5, 2, 2.5, and 3) values with 5 M HCl. After incubation for 30 min at 37°C, 1 ml of the mixture was removed to determine viable counts (expressed as CFU/ml) on MRS agar taking as reference the concentration of bacteria not exposed to simulated gastric fluid. *Lactobacillus rhamnosus* GG (lab collection) was used as a positive control because it is a probiotic bacterium well known for its resistance to gastrointestinal conditions.

### β-Galactosidase Activity

The LAB strains were grown in MRS broth at 37°C for 24 h, streaked onto MRS agar and incubated at 37°C for 48 h. One colony was transferred to a tube containing a disk of *O*-nitrophenyl-β-D-galactopyranoside—ONGP (Sigma–Aldrich) and 100 μl sterile saline (0.85% NaCl). A yellow color indicated the release of o-nitrophenol (chromogenic compound) and represented a positive result for the production of β-galactosidase.

### Hemolytic Activity

Testing for hemolytic activity was carried out as described by Carrillo et al. (1996). Isolated strains were screened for hemolytic activity on blood agar plates containing 5% (v/v) horse blood and incubated at 30°C for 24–48 h. A clear zone around the colony indicated hemolytic activity, which was probably caused by surfactant production. The zones of clearing were scored as follows: (-) no hemolysis; (+) incomplete hemolysis, when the zone was not totally clear; (++) complete hemolysis with a diameter of lysis < 1 cm; (+++) complete hemolysis with a diameter of lysis between 1 cm and 3 cm; and (++++) complete hemolysis with a diameter of lysis > 3 cm.

### Drop Collapse Test

The drop collapse test was carried out as described by Jain et al. (1991). LAB were cultivated in MRS at 37°C for 24 h, centrifuged at 12,000 × *g* for 5 min and 100 μl of the supernatants were added to each well of 96-well microplates (TPP, Switzerland) and then 5 μl of crude motor oil was added to the surface. A result was considered positive for biosurfactant production when the drop diameter was at least 1 mm larger than that produced by deionized water (negative control). Each test was repeated in two separate microtiter plates.

### Microbial Adhesion to Hydrocarbon Test (MATH)

Bacterial cell surface hydrophobicity was assessed by measuring adhesion to hydrocarbons (MATH) as described by Kotzamanidis et al. (2010). LAB cultivated in MRS at 37°C for 24 h were washed twice in phosphate-buffered saline (PBS; 10 mM Na_2_HPO_4_, 1 mM KH_2_PO_4_, 140 mM NaCl, 3 mM KCl) and re-suspended in 3 mL of 0.1 M KNO_3_ to achieve approximately 10^8^ CFU/ml (OD_600__nm_ = 0.2). Absorbance of the suspension was measured at 600 nm (*A*_0_). One microliter of xylene was added to the cell suspension to form a two-phase system and after 10 min at room temperature, the two-phase system was mixed by vortexing for 2 min. After 20 min at room temperature (approximately 23°C), the aqueous phase was carefully removed and absorbance at 600 nm (*A*_1_) measured. The percentage of cell surface hydrophobicity (H, %) was calculated using the following formula: H (%) = (1 *A*_1_/*A*_0_) ^∗^ 100, where *A*_1_ represents the absorbance of the mixture after 20 min at room temperature and *A*_0_, absorbance at time 0.

### Gelatinase Activity

Gelatinase production was verified by spotting 1 μl aliquots of the 24 h cultures onto the surface of five Luria Bertani agar plates (BD, Franklin Lakes, NJ, USA) supplemented with 3% (w/v) gelatin (BD). Plates were incubated at 37°C and 42°C for 48 h, 25°C for 72 h, and 10°C and 15°C for 10 days. After incubation, the plates were maintained at 4°C for 4 h and the hydrolysis of gelatin was recorded by the formation of opaque halos around the colonies (Perin et al., 2014).

### Antibiotic Resistance

The resistance to antibiotics was determined by the broth microdilution protocol according to Muñoz et al. (2014) with some modifications. Antibiotics employed in this study were β-lactams (ampicillin: AMP), quinolone (ciprofloxacin: CIP), lincosamide (clindamycin: CLI), aminoglycosides (gentamicin: GEN, kanamycin: KAN and streptomycin: STR), macrolides (erythromycin: ERY), glycopeptides (vancomycin: VAN), chloramphenicol: CMP and tetracycline: TET. These antibiotics were selected based on the European Food Safety Authority recommendations for probiotics strains (European Food Safety Authority [EFSA], 2012). All antibiotics were purchased from Sigma–Aldrich, USA. To prepare the stock antibiotic solutions, each antibiotic was weighed, dissolved in sterile distilled water (except CMP which was dissolved in sterile distilled water with 0.5% of ethanol), filter-sterilized (0.2 mm) and kept at -20°C until use. The working solutions at specific concentrations were prepared daily. Overnight cultures were adjusted to OD_600__nm_ of 0.8 (10^9^ CFU/ml) with PBS, and used to inoculate (1% v/v) Mueller Hinton broth (Oxoid, Basingstoke, England) containing each antibiotic at tested concentrations (final volume of 100 μl per well of 96 micro-well plates). The plates were incubated at 37°C for 24 h. Resistance rates were calculated according to microbial cut-off values (mg/ml), as recommended by the European Food Safety Authority [EFSA] (2012). The microbiological breakpoints were defined according to Danielsen and Wind (2003), Flórez et al. (2005) and the European Commission (European Commission SCAN, 2007).

### Virulence Genes

Total DNA extraction was performed using a Blood and Tissue mini kit Quiagen (German Town, USA). The primers used for the amplification of genes *esp, agg, gelE, efaAfm* and *efaAfs, cylA, cylB* and *cylM* were described by Eaton and Gasson (2001), and primers of *cyl* operon (*cylLL* and *cylLS*) were developed by Semedo et al. (2003). **Table 2** describes the primers used in these tests. All primers were synthesized by Life Technology (Brazil). PCR amplifications were performed in a ThermoCycler AB (Applied Biosystems Veriti, NJ, USA), in 0.2-ml reaction tubes containing 25 μl of GoTaq^®^ Green Master Mix, 2.5 μ1 (10 μM) of each primer, and 1 μl (100 ng) of DNA. Amplification reactions were as follows: initial cycle of 94°C for 1 min, 35 cycles of 94°C for 1 min, 55°C for 1 min, 72°C for 2 min, a final extension step of 72°C for 7 min and then cooling to 4°C. Amplification products were submitted to electrophoresis in 1% (w/v) agarose gel at 100 V for 30 min. A 100-bp PCR DNA ladder was used as the molecular weight marker. The gels were photographed on a Gel Doc^TM^ XR+ System (BioRad, Richmond, CA, USA), and image analysis was accomplished using Quantity One software. The positive control was *Enterococcus faecalis* FI 9190 (obtained from Eaton and Gasson, 2001, Institute of Food Research, Norwich Research Park, Norwich, UK). For each PCR, a negative control (sample without template) was included.

**Table 2:** Primers used to test for the presence of virulence genes.

Target gene^∗^	Primers	Fragment size (pb)
*Agg*	AAGAAAAAGAAGTAGACCAAC AAACGGCAAGACAAGTAAATA	1,553
*GelE*	ACCCCGTATCATTGGTTT ACGCATTGCTTTTCCATC	419
*esp*	TTGCTAATGCTACTCCACGACC GCGTCAACACTTGCATTGCCGAA	933
*efaAfs*	GACAGACCCTCACGAATA AGTTCATCATGCTGTAGTA	705
*efaAfm*	AACAGATCCGCATGAATA CATTTCATCATCTGATAGTA	735
*cpd*	TGGTGGGTTATTTTTCAATTC TACGGCTCTGGCTTACTA	782
*Cob*	AACATTCAGCAAACAAAGC TTGTCATAAAGAGTGGTCAT	1,405
*Ccf*	GGGAATTGAGTAGTGAAGAAG AGCCGCTAAAATCGGTAAAAT	543
*cylLL*	GATGGAGGGTAAGAATTATGG GCTTCACCTCACTAAGTTTTATAG	253
*cylLS*	GAAGCACAGTGCTAAATAAGG GTATAAGAGGGCTAGTTTCAC	240
*cylM*	AAAAGGAGTGCTTACATGGAAGAT CATAACCCACACCACTGATTCC	2,940
*cylB*	AAGTACACTAGTACAACTAAGGGA ACAGTGAACGATATAACTCGCTATT	2,020
*CylA*	TAGCGAGTTATATCGTTCACTGTA CTCACCTCTTTGTATTTAAGCATG	1,282

*^∗^*Agg* (Aggregation protein), *gelE* (gelatinase), *esp* (cell-wall-associated protein) *efaAfm* and *efaAfs* (cell wall adhesins*), cpd, cob* and *ccf* (sex pheromones, chemotactic for human leukocytes, facilitate conjugation), *cylLL* and *cylLS* (Cytolysin precursor), *cylM* (post-translational modification of cytolysin), *cylB* (transport of cytolysin) *and cylA* (activation of cytolysin).*

### Biofilm Assay

The quantification of biofilm production was performed as described previously by Borges et al. (2012) with some modifications. The wells of a sterile 12-well polystyrene microtiter plate (TPP, Switzerland) were filled with 2 ml of MRS broth, absorbance (*A*_600__nm_) of bacterial suspensions in MRS was adjusted to 0.25 ± 0.05 in order to standardize the number of bacteria (10^7^–10^8^ CFU/ml) and 200 μl of overnight was added to each well. The plates were incubated aerobically for 48 h at 30°C. To quantify the biofilm formation, the wells were gently washed three times with 2 ml of sterile distilled water. The attached bacteria were fixed with 2 ml of methanol (Romyl, Leics, UK) for 15 min, and then, microplates were emptied and dried at room temperature. Subsequently, 2 ml of a 2% (v/v) crystal violet solution was added to each well and held at ambient temperature for 5 min. Excess stain was then removed by placing the plate under gently running tap water. Stain was released from adherent cells with 2 ml of 33% (v/v) glacial acetic acid. The optical density (OD) of each well was measured at 595 nm using a plate reader (Microplate reader, Bio-Rad, Hercules; CA, USA). Each assay was performed in four replicates and conducted three individual times on different days under the same conditions, and the negative control was performed in uninoculated MRS broth. The cut-off (ODC) was defined as the mean OD value of the negative control. Based on the OD, strains were classified as non-biofilm producers (OD ≤ ODC), weak (ODC < OD ≤ 2 × ODC), moderate (2 × ODC < OD ≤ 4 × ODC) or strong biofilm producers (4 × ODC < OD; Borges et al., 2012).

### Inhibition of Biofilm Formation

Lactic acid bacteria strains were inoculated (1% v/v) in 2 ml of MRS broth diluted to one-fifth of the concentration recommended by the manufacturer (55 g/l) and transferred (2 ml/well) to 12-well polystyrene microtiter plates (TPP, Switzerland). The plates were incubated at 30°C for 48 h for attachment of cells to the wells (biofilm formation). The broths were carefully discarded by pipetting and the biofilms visually present on the bottom and sides of the plate were washed with 2 ml PBS pH 7.1 (10 mM Na_2_HPO_4_, 1 mM KH_2_PO_4_, 140 mM NaCl, 3 mM KCl) to remove planktonic and loosely attached cells. Absorbance (*A*_600__nm_) of pathogenic bacterial suspensions in TSB was adjusted to 0.25 ± 0.05 in order to standardize the number of bacteria (10^7^–10^8^ CFU/ml), added to biofilms and incubated at 30°C for 24, 48, and 72h. Every 24 h, half of the broth in the wells was replaced with fresh broth. After incubation, the planktonic cultures were carefully removed and the biofilms were suspended by scrapping and vigorous shaking.. To evaluate the viable count of adherent microorganisms in the biofilm, three wells for each strain were washed three times as previously described and scraped. The obtained suspensions were transferred into sterile tubes and mixed with a vortex mixer for 30 s. Proper dilutions were prepared in saline solution 0.85% (w/v) and plated on xylose lysine deoxycholate agar (XLD) for *S.* Typhimurium, Modified Oxford agar (MOX) for *Listeria monocytogenes* and MacConkey sorbitol agar (SM) for *E. coli* O157:H7. The plates were incubated at 37°C for 24–48 h and bacterial counts were performed.

*Listeria monocytogenes, S.* Typhimurium, and *E. coli* O157:H7 controls were used to monitor the biofilm development of these strains without the presence of LAB biofilms.

Pathogenic planktonic cells counts were performed from the broths discarded by pipetting, following the same procedures used for biofilm cell count (data not shown).

### Statistical Analysis

All experiments were carried out three times, with duplicate samples per trial, and results were expressed as average. Standard deviations were determined with Excel programme (Microsoft Corp., USA). A *t*-test was performed at the 95% confidence interval with PASW Statistics—SPSS 17 (IBM Co.), in order to determine the statistical significance of data.

## Results

### Auto-Aggregation and Co-Aggregation Assays

**FIGURE 1:**
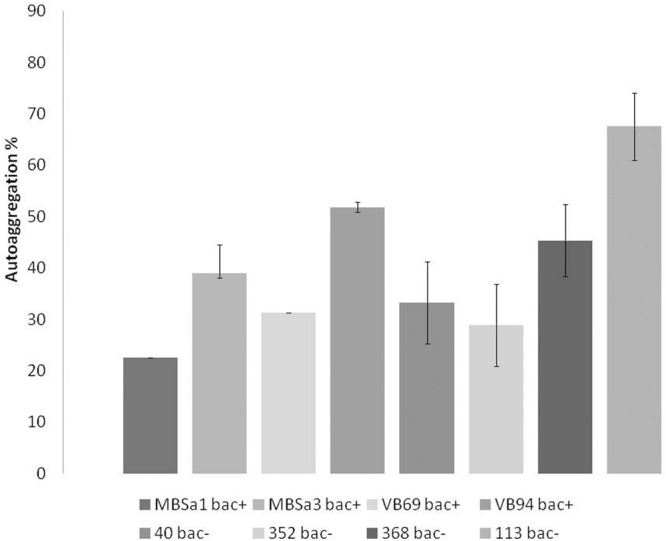
**Auto-aggregation of lactic acid bacteria strains cells re-suspended in PBS (pH 7.1) evaluated after 24 h incubation at 37°C.** Error bars represent standard deviations of the mean values of results from three replicated experiments (bac^+^ = bacteriocin producer).

**FIGURE 2:**
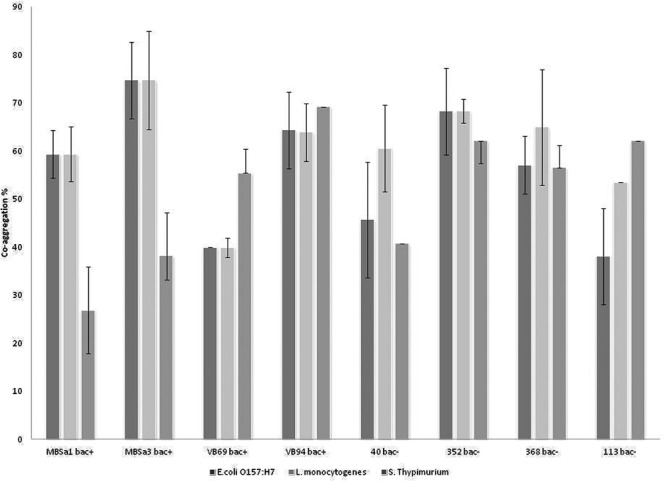
**Co-aggregation values recorded for lactic acid bacteria strains with *Listeria monocytogenes* ATCC 7644, *Salmonella* Typhimurium ATCC 14028, and *Escherichia coli* O157:H7 ATCC 35150 after 24 h incubation at 37°C in PBS (pH 7.1).** Error bars represent standard deviations of the mean values of results from three replicate experiments (bac^+^ = bacteriocin producer).

Aggregation values increased over time in a strain-dependent manner. *W. viridescens* 113 presented the highest auto-aggregation (67%), compared to the other isolates showing only moderate auto-aggregation (**Figure 1**). All LAB strains presented co-aggregation with pathogens (**Figure 2**), in a strain–pathogen combination-dependent manner. *Lactobacillus curvatus* MBSa3 exhibited the highest co-aggregation (69% with *Listeria monocytogenes* and 74.6% with *E. coli* O157:H7), while the lowest co-aggregation was exhibited by *W. viridescens* 113 (53.4% with *Listeria monocytogenes* and 38% with *E. coli* O157:H7).

### Tolerance to Bile Salts and Acidic pH

**FIGURE 3:**
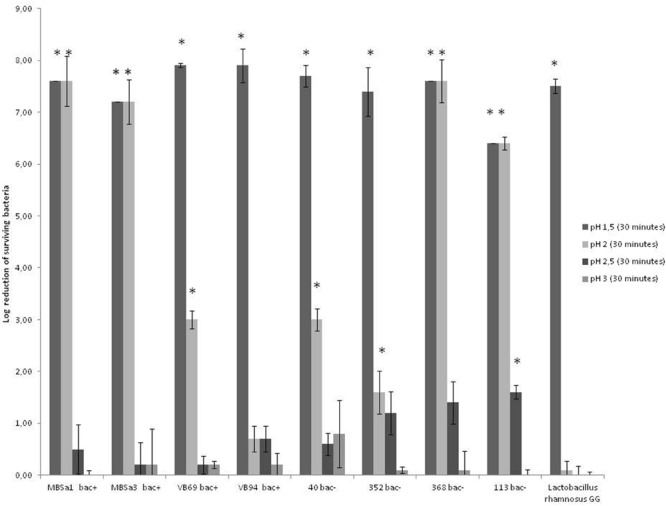
**Log reduction of lactic acid bacteria strains after incubation at 37°C for 30 min in (SFG) Simulated Grastric Fluid; 3.2 g/l pepsin and 2 g/l NaCl (pH 1.5–3).** Log reductions were estimated by subtracting the log of surviving to the controls (bacteria not exposed to simulated gastric fluid). Data are mean ± standard deviations. Superscript ^∗^ indicates a significant difference (*p* < 0.05) compared to the control. Detection limit is 10 CFU/ml.

The results showed that tolerance for bile salts mixture from Sigma was 4% for all LAB strains. However, the tolerance to bile salts from Oxoid was 20% for *W. viridescens 113* and *L. lactis* 94 and 8% for *Lactobacillus casei* 40 and *L. lactis* 69 (data not shown) for the rest of studied strains was 4%. The results in **Figure 3** show that all tested strains, including *Lactobacillus rhamnosus* GG, survived to exposure to pH 2.5 for 30 min. No significant difference (*p* < 0.05) between the initial microbial population and the population after 30 min at pH 2.5 was observed for all strains; a reduction of viability was only observed for *W. viridescens* 113, approximately 2 log. However, a significant reduction of viability at pH 2.0 was observed for all tested bacteria except for *L. lactis* 94. In counterpart, complete survival at pH 3 and no survival at pH 1.5 were observed for all strains.

### Biosurfactant Production

The three screening tests indicated that all tested LAB strains were capable to produce biosurfactant (**Table 3**). In the hemolysis test, most strains showed zones of clearing in the blood agar with scores corresponding to (++) indicating complete hemolysis with a diameter < 1 cm. The exception was *L. lactis* 94 that was not hemolytic. In the MATH assay, the lowest values observed were 77.2% (*Lactobacillus casei* 40); 76.4% (*Lactobacillus curvatus* MBSa3); 81.2% (*L. lactis* 368) and 88.9% (*Lactobacillus sakei* MBSa1) for the rest of LAB studied the values was over 90% with 91.2% (*Lactobacillus helveticus* 352); 93.9% (*W. viridescens* 113); 95.1% (*L. lactis* 94) and 95.2% (*L. lactis* 69).

**Table 3:** Biosurfactant production by the LAB strains.

Method			
Strains	Hemolysis in blood agar^a^	Drop collapse test^b^	MATH test^c^ (%)
*Lactobacillus sakei* MBSa1 bac+	++	++	88.9 ± 0.14
*Lactobacillus curvatus* MBSa3 bac+	++	++	76.4 ± 0.23
*Lactococcus lactis* VB69 bac+	+	++	95.2 ± 0.09
*Lactococcus lactis* VB94 bac+	–	+	95.1 ± 0.13
*Lactobacillus casei* 40 bac–	+	+	77.2 ± 0.30
*Lactobacillus helveticus* 352 bac–	+	+	91.2 ± 0.4
*Lactococcus lactis* 368 bac–	++	++	81.2 ± 0.11
*Weissella virisdescens* 113 bac–	++	++	93.9 ± 0.32

*^a^(+) incomplete hemolysis; (++) complete hemolysis with a diameter of lysis < 1 cm; (+++) complete hemolysis with a diameter of lysis > 1 cm but < 3 cm; and (++++) complete hemolysis with a diameter of lysis > 3 cm and green colonies.*

*^b^Flat drops with scoring system ranging from + to ++++ corresponding to partial to complete spreading on the oil Surface. Rounded drops were scored as negative—indicative of the lack of biosurfactant production.*

*^c^Percent of bacterial cell surface hydrophobicity.*

All strains resulted positive in the drop collapse test. Flat drops with scoring system ranging from + to ++++ corresponding to partial to complete spreading on the oil surface. The strains studied did not present complete spreading on the oil surface only a partial spreading was observed, varying between + for *L. lactis* 94, *Lactobacillus casei* 40, and *Lactobacillus helveticus* 352 to ++ in the rest of strains studied, *L. lactis* 69, *W. viridescens* 113 *Lactobacillus sakei* MBSa1 and *Lactobacillus curvatus* MBSa3.

### Antibiotic Resistance, Presence of Virulence Genes and Gelatinase Activity

The antibiotic susceptibility tests (**Table 4**) indicated that the MIC for ciprofloxacin, clindamycin, gentamicin, kanamycin, and streptomycin did not exceed the epidemiological cut-off suggested by the European Food Safety Authority [EFSA] (2012) for all tested strains. All strains were sensitive to β-lactams (ampicillin: AMP), except *Lactobacillus curvatus* MBSa1. Some strains were resistant to one or more antibiotics: *Lactobacillus casei* 40 and *Lactobacillus curvatus* MBSa1 were resistant to erythromycin, *Lactobacillus sakei* MBSa3 and *Lactobacillus casei* 40 were resistant to chloramphenicol; *Lactobacillus curvatus* MBSa1, *L. lactis* 94 and 368 were resistant to vancomycin. Only *Lactobacillus casei* 40, *Lactobacillus helveticus* 352 and *L. lactis* 69 were sensitive to tetracycline. All strains were sensitive to erythromycin, except *Lactobacillus casei* 40 and *Lactobacillus curvatus* MBSa1.

**Table 4:** Determination of minimal inhibitory concentration (MIC) against the LAB strains.

Strains	MICs (μg/ml)									
	CIP	CMP	VAN	ERY	STR	CLI	GEN	AMP	TET	KAN
*Lactobacillus sakei* MBSa1 bac+	< 0.5	1	** > 20**	**5**	5	< 0.5	1	**5**	**10**	< 0.5
*Lactobacillus curvatus* MBSa3 bac+	20	** > 20**	< 0.5	10	10	< 0.5	<0.5	< 0.5	**10**	< 0.5
*Lactococcus lactis* VB69 bac+	1	10	10	5	5	< 0.5	5	1	5	5
*Lactobacillus casei* 40 bac-	1	** > 20**	>20n.r	** > 20**	20	< 0.5	1	0.5	< 0.5	0.5
*Lactobacillus helveticus* 352 bac-	< 0.5	<0.5	< 0.5	5	5	< 0.5	1	0.5	< 0.5	<0.5
*Lactococcus lactis* 368 bac-	1	10	** > 20**	10	10	< 0.5	5	10	**10**	< 0.5
*Weissella virisdescens* 113 bac-	< 0.5	1	<0.5	1	1	< 0.25	1	0.5	**20**	< 0.5

*CIP, ciprofloxacin; CMP, chloramphenicol; VAN, vancomycin; ERY, erythromycin, STR, streptomycin; CLI, clindamycin; GEN, gentamicin; AMP, ampicillin; TET, tetracycline; and KAN, kanamycin.*

*Resistant strains with an MIC value higher than the breakpoints described in the table are indicated in bold.*

*nr, not required.*

**Table 5** shows the presence of the virulence genes tested in the LAB strains. All strains were negative for *GelE, cob, ccf, cylLL, cylLs, cyllM, cylB, cylA* and *efaAfs*, except *W. viridescens* 113 that was positive for *cob* and for *GelE*. Nevertheless, no strain presented gelatinase activity. *Lactobacillus helveticus* 352 was positive for *Agg* and *efaAfm, L. lactis* 368 for *cpd* and *efaAfm* too and the presence of *esp* was observed in *L. lactis* 94 and 69, *Lactobacillus casei* 40, and *Lactobacillus curvatus* MBSa3.

**Table 5:** Presence of virulence genes in the LAB strains.

Strain	Genes												
	*Agg*	*GelE*	*esp*	*efaAfs*	*efaAfm*	*cpd*	*cob*	*ccf*	*cylLL*	*cylLS*	*cylM*	*cylB*	*CylA*
*Lactobacillus sakei* MBSa1 bac+	–	–	–	–	+	–	–	–	–	–	–	–	–
*Lactobacillus curvatus* MBSa3 bac+	–	–	+	–	+	–	–	–	–	–	–	–	–
*Lactococcus lactis* VB69 bac+	–	–	+	–	+	–	–	–	–	–	–	–	–
*Lactococcus lactis* VB94 bac+	–	–	+	-	+	–	–	–	–	–	–	–	–
*Lactobacillus casei* 40 bac–	–	–	+	–	+	–	–	–	–	–	–	–	–
*Lactobacillus helveticus* 352 bac–	+	–	–	–	–	+	–	–	–	–	–	–	–
*Lactococcus lactis* 368 bac–	–	–	–	–	–	+	–	–	–	–	–	–	–
*Weissella virisdescens* 113 bac–	–	+	–	–	+	–	+	–	–	–	–	–	–

### Biofilm Assay

**FIGURE 4:**
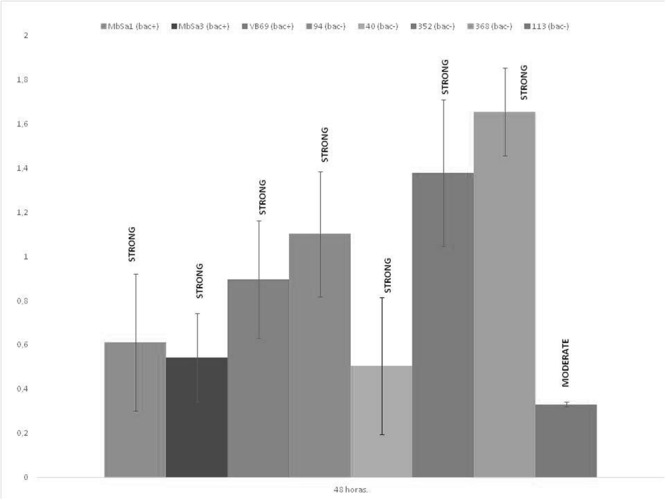
**Biofilm formation of LAB using the microtiter plate assay.** After incubation at 30°C for 48 h in MRS media. Data are mean ± standard deviations. The cut-off (ODC) was defined as the mean OD value of the negative control. Based on the OD, strains were classified as non-biofilm producers (OD ≤ ODC), weak (ODC < OD ≤ 2 × ODC), moderate (2 × ODC < OD 4 × ODC) or strong biofilm producers (4 × ODC < OD).

All the strains studies were biofilm producers in MRS. The biofilm production was strain dependent (**Figure 4**). Based on the OD, all the strains studied were strong producer’s except *W. viridescens* 113. The highest values over 1, were observed for *L. lactis* 368 (1.65), *Lactobacillus helveticus* 352 (1.38) and *L. lactis* 94 (1.10). The values for the rest of strains were under 1, but all were strong biofilm producers except *W. viridescens* 113 with moderate biofilm formation.

### Inhibition of Biofilm Formation

**FIGURE 5:**
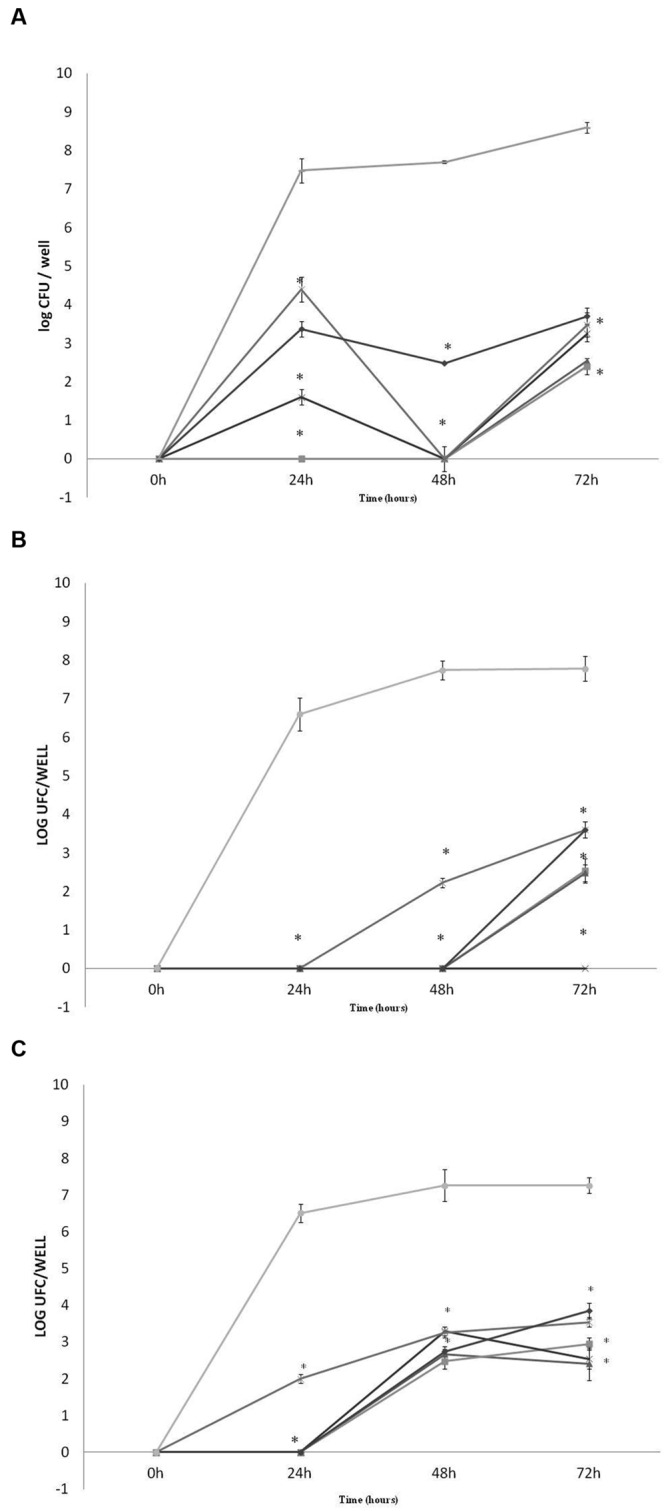
**Quantification of pathogen biofilms on microtiter plates in MRS broth (**A**, *Listeria monocytogenes* ATCC 7644, **B**, *S.* Typhimurium ATCC 14028, **C**, *E. coli* O157:H7 ATCC 35150) in the presence of *W. viridescens* 113 bac- (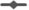); *L. lactis* 69 bac+ (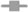); *L. lactis* 94 bac+ (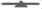); *Lactobacillus casei* 40 bac- (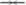), and *Lactobacillus helveticus* 352 bac- (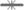), biofilms after 24, 48, and 72h at 30°C.** *Listeria monocytogenes, S.* Typhimurium, and *E. coli* O157:H7 positive control (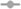). No counts of the pathogens biofilms were detected in the presence of *Lactobacillus. sakei* MBSa1 bac+, *Lactobacillus curvatus* MBSa3 bac+ and *L. lactis* 368 bac- biofilms. Results are mean of triplicates and vertical bars show standard deviations. Superscript ^∗^ indicates a significant difference (*p* < 0.05) compared to the control (pathogens alone).

The total inhibition in pathogens *E. coli* O157:H7, *Listeria monocytogenes* and *S.* Typhimurium biofilm formation, in 24, 48, and 72 h of exposure, was obtained for *L. lactis* 368 (bac-), *Lactobacillus curvatus* MBSa3 (bac+) and *Lactobacillus sakei* MBSa1 (bac+). For the other strains, the inhibition was time-dependent and varied according to the strain and target pathogen (**Figure 5**). The presence of sessile cells of *E. coli* O157:H7, *Listeria monocytogenes* and *S.* Typhimurium in the presence of LAB in 24, 48, and 72 h was significantly reduced in comparison to the pure cultures (*p* < 0.05). *Listeria monocytogenes* was not detected within *L. lactis* 69 (bac+) and 94 (bac+) established biofilms following 24 h and 48h interaction periods. Nevertheless, the presence of *Listeria monocytogenes* biofilms were observed in the cases of *W. viridescens* 113 (bac-), *Lactobacillus casei* 40 (bac-) and *Lactobacillus helveticus* 352 (bac-); 4 log of decrease was observed for 24 h of incubation in presence of *Lactobacillus helveticus* 352 (bac-) biofilm, as well as, 7 log of decrease for *Lactobacillus casei* 40 (bac-) during the same incubation time. After 48 h of incubation 5 log of decrease were detected in the presence of *W. viridescens* 113 (bac-). The presence of *Listeria monocytogenes* biofilms was detected during 72 h of incubation in all cases, varying between 4 log for *W. viridescens* 113 (bac-), *Lactobacillus helveticus* 352 (bac-) and *Lactobacillus casei* 40 (bac-) to 6 log of decrease in the cases of *L. lactis* 94 (bac+) and 69 (bac+). In *S.* Typhimurium experiment, sessile cells were not detected during 24 h of incubation in the presence of most LAB tested, only for *Lactobacillus helveticus* 352 (bac-) 2 log were achieved (6 log of decrease). After 48 and 72 h only in the presence of *Lactobacillus casei* 40 (bac-) sessile cells of *S.* Typhimurium were not detected. For *E. coli* O157:H7 only after 24 h of incubation the presence was not detected, except for *Lactobacillus helveticus* 352 (bac-). During 48 and 72 h approximately 3 log of *E. coli* was detected (5 log of decrease) in the presence of all tested LAB. In most cases, reductions between 5 and 3 log for *E. coli* O157:H7, 4log for *S.* Typhimurium and between 7 and 3 log for *Listeria monocytogenes* were achieved.

In addition, when supernatants were studied, planktonic pathogens cells were not detected, in all studied cases counts of pathogenic cells were below the detection limit (<10 CFU/ml, data not shown).

## Discussion

The increased resistance to disinfection processes may be aggravated when bacterial biofilms are formed on surfaces that are recalcitrant for clean, such as cracks, holes, or tube connections. When planktonic cells are released from these colonization microenvironments, they may enter the food production chain and proliferate if proper conditions for growth occur, compromising the safety, quality, and stability of the final product. The application of the competitive biofilms formed by bacteria that produce natural antimicrobial substances and biosurfactants can provide new opportunities for the control of pathogenic bacteria and avoid food cross contamination.

Aggregation and co-aggregation among bacteria play an important role in prevention of colonization of surfaces by pathogens (García-Cayuela et al., 2014) as it is well known that co-aggregation abilities of LAB strains might interfere with the ability of the pathogenic species to infect the host and can prevent the colonization of food-borne pathogens (García-Cayuela et al., 2014). In this study, the tested LAB, especially the bacteriocin-producing *Lactobacillus* strains, presented high auto-aggregation and co-aggregation results, *Lactobacillus curvatus* MBSa3 exhibited the highest co-aggregation (69% with *Listeria monocytogenes* and 74.6% with *E. coli* O157:H7) and in this case pathogenic biofilms were not detected after three times of incubation tested, 24, 48, and 72; in other side the lowest co-aggregation was exhibited by *W. viridescens* 113 (53.4% with *Listeria monocytogenes* and 38% with *E. coli* O157:H7) and pathogenic cells were detected in 48 and 72 h of incubation in the presence of biofilm from strain. Nevertheless in other strains, there was apparently no relationship between the detection of pathogens and the percentage of co-aggregation with them.

Aggregation can also increase the concentration of excreted inhibitory substances (Kaewnopparat et al., 2013). Thus, these food-associated lactobacilli that co-aggregate numerous pathogens are of special interest with regard to potential applications in food-processing plants. Correlation between adhesion ability and hydrophobicity, as measured by microbial adhesion to hydrocarbons, has been reported for some lactobacilli (Wadström et al., 1987), but also conflicting results have been reported (Vinderola et al., 2004). As a result, adhesion, surface hydrophobicity, autoaggregation, and co-aggregation are phenotypic traits that potentially provide microbial colonization advantages within the intestinal tract. Aggregation abilities and cell surface hydrophobicity may not be the only components responsible for adhesion but these are some of the criteria to bear in mind of a complex mechanism that enables microorganisms to interact with the host and exert its beneficial effect (García-Cayuela et al., 2014).

The result obtained in hydrophobicity, aggregation, and co-aggregaton tests correspond with previus works like García-Cayuela et al. (2014). Di Bonaventura et al. (2008) reported a connection between hydrophobicity of cell surface and bacterial attachment, colonization, and biofilm formation. Our results show high values of hydrophobicity as well as a strong biofilm production, for most of the strains studied but there was no apparent correlation between hydrophobicity highest values and the strongest biofilm production. *W. viridescens* 113 shows a moderate biofilm production while displaying one of the highest hydrophobicity values. All tested LAB strains were tolerant to bile salts and acidic pH, evidencing their resistance to digestive stress and potential as probiotic agents. For a probiotic microorganism to be of benefit to human health it must survive the passage through the upper GIT and be able to function in the gut environment (Giraffa et al., 2010). Their functional requirements include tolerance to acid and bile, adherence to epithelial surfaces and antagonistic activity toward intestinal pathogens (Ramos et al., 2013; Peres et al., 2014). All LAB strains except *L. lactis* 368 were negative for β-galactosidase production (data not shown). This characteristic is disadvantageous for the probiotic activity of most studied LAB, as strains able to hydrolyze lactose might be useful for minimizing the effects of lactose intolerance (De Vrese et al., 2001).

Resistance of the LAB strains to antibiotics was species and strain dependent. *Lactobacillus helveticus* 352 and *L. lactis* VB69 were susceptible to all tested antibiotics, but *Lactobacillus sakei* MBSa1 was resistant to vancomycin, erythromycin, ampicillin, and tetracycline. Data from various studies on *Lactobacillus* spp. resistance to various antimicrobial agents demonstrate the existence of inter-genus and inter-species differences (Danielsen and Wind, 2003). The natural resistance to multiple classes of antibiotics is probably due to cell wall structure and membrane permeability, complemented in some cases by the eﬄux mechanisms (Ammor et al., 2007). However, this feature might represent a competitive advantage, especially when a probiotic product is administered with antimicrobials for treatment of an infectious disease thereby reducing the likelihood of disbiosis (microbial imbalance), rapidly rebalancing normal microbiota (Peres et al., 2014). The EFSA requires that bacteria which are to be introduced into the food chain lack acquired antimicrobial resistance determinants to prevent lateral spread of these (van Reenen and Dicks, 2011). Therefore for the cases of strains who presented antibiotic resistances, future genetic studies are needed to confirm if this resistance is due to acquired antimicrobials determinants. The presence of *efaAfm* in some strains seems to have no value as a risk indicator since this gene was also found in starter *E. faecium* strains with a long record of safe use in food (Eaton and Gasson, 2001). High frequencies of positive results were observed for, *esp* and *efaAfm*, in *Lactococcus* and *Lactobacillus* strains (**Table 5**). Furthermore, *efaAfm* and *esp* genes are related to the production of substances enrolled in the microbial colonization and adhesion at biotic and non-biotic surfaces (Valenzuela et al., 2009). *W. viridescens* 113 was positive for *GelE* but did not produce gelatinase, Eaton and Gasson (2001) described that *gelE* expression is highly influenced by the culture conditions, and the laboratory manipulation of the strains can result in the loss of the structural genes, and can explain the loss of gelatinase activity during *in vitro* tests. Moreover, *W. virisdescens* 113 and *L. lactis* 368 were positive for *cob* and *cpd* genes respectively, which are related to sex pheromones, although sex pheromones are not considered *per se* as virulence factors (Valenzuela et al., 2008). No strain was found positive for cytolisin family genes, and this confirms that the hemolysis present in blood agar was not related with these virulent genes.

Biosurfactant production is an interesting character, which can be related to the inhibition of the attachment of pathogens. The anti-adhesive and anti-biofilm-forming properties of lactobacilli have been reported in previous studies, such as *Lactobacillus delbrueckii* against *E. coli* (Abedi et al., 2013) and *Lactobacillus brevis* CD2 against *Prevotella melaninogenica* (Vuotto et al., 2013). In addition, *Lactobacillus* species were able to displace adhering uropathogenic *Enterococcus faecalis* from hydrophobic and hydrophilic substrata in a parallel-plate flow chamber (Velraeds et al., 1996). Biosurfactants from LABs have been shown to reduce adhesion of bacterial pathogens to glass, silicone rubber, surgical implants, and voice prostheses (Rodrigues et al., 2004). One xylolipid biosurfactant produced by a *L. lactis* strain with broad antibacterial activity against multidrug resistant *E. coli* and *Staphylococcus aureus* was described (Saravanakumari and Mani, 2010). Biosurfactants also been reported to have strong antifungal and antiviral activity (Singh and Cameotra, 2004). For the screening in biosurfactant production by haemolytic test, all the strains were positive except *L. lactis* 94. The strains showed, complete hemolysis with a diameter of lysis < 1 cm. In addition, drop collapse test was positive for all tested strains corresponding with partial spreading on the oil surface. None of the studies reported in the literature (Johnson and Boese-Marrazzo, 1980; Banat, 1993; Carrillo et al., 1996; Morán et al., 2002) mention the possibility of biosurfactant production without a hemolytic activity. However, in some cases hemolytic assay excluded many good biosurfactant producers (Youssef et al., 2004); hence in the present investigation the MATH assay and drop collapse test with crude oil were also done to confirm biosurfactant production.

The results of this study indicate that the tested LAB was capable to reduce *Listeria monocytogenes, Salmonella* and *E. coli* O157:H7 biofilm formation, and present probiotic characteristics and potentially no risk for the consumers. All strains were capable to hinder the development of pathogens in the first 72 h of incubation. Woo and Ahn (2013) obtained similar results of *Listeria monocytogenes* and *Salmonella* inhibition testing probiotic strains. Kim et al. (2013) showed the inactivation of *E. coli* O157:H7 on stainless steel upon exposure to *Paenibacillus polymyxa* biofilms. Zhao et al. (2013) reported the reduction of *Listeria monocytogenes* in a ready-to-eat poultry processing plant by LAB and Pérez-Ibarreche et al. (2014) reported that lactobacilli with biofilm-forming aptitudes were able to control *Listeria monocytogenes* biofilms. In this study inhibition, effect against biofilm adhesion was observed in bacteriocin producers *L. lactis* VB69 and VB94; *Lactobacillus sakei* MBSa1 and *Lactobacillus curvatus* MBSa3 as well as non-bacteriocin producers *Lactococcus lactis—lactis* 368, *Lactobacillus helveticus* 354, *Lactobacillus casei* 40 and *W. viridescens* 113. It seems that inhibition of pathogenic bacteria growth and adhesion is not only due to the bacteriocin production. This outcome can be attributed to a combination of factors like biosurfactant and bacteriocin production as well as mechanisms of pathogens exclusion through their trapping (killing of cells embedded in biofilms). This is in accordance with previous works like Guerrieri et al. (2009) witch suggests the need to apply the bacteriocine-producing microorganism, in biofilms. There may be an influence of EPS (exo-polysaccharide). Kim et al. (2006) found that the EPS of *Lactobacillus acidophilus* A4 had stronger anti-biofilm activity against the growth of entero-hemorrhagic *E. coli* O157: H7, *S. enteritidis, S. typhimurium* KKCCM 11806, *Yersinia enterocolitica, Pseudomonas aeruginosa* KCCM 11321, *Listeria monocytogenes* Scott A, and *B. cereus*.

## Conclusion

Our results show that LAB strains from foods can be excellent candidates to form protective biofilms, in accordance with the hypothesis proposed by Falagas and Makris (2009) to use non-pathogenic microorganisms, namely probiotics, as part of daily cleaning products to lower the incidence of pathogenic microorganisms. Evidences on the efficacy of probiotics for the prevention and treatment of infections have been observed both *in vitro* and *in vivo* (Levkovich et al., 2013; Shu et al., 2013). The present study provided new information about the use of potential probiotic LAB biofilms for the control of *Listeria monocytogenes, S.* Typhimurium and *E. coli* O157:H7 biofilms formation through exclusion mechanisms. However, more experiments are needed to confirm the ability of these strains to inhibit the pathogen biofilm formation in other environments. Our initial studies are very encouraging and indicate that the LAB that we have tested are promising candidates for controlling the presence of pathogenic biofilms in food-processing facilities. The development of protective biofilms with probiotic LAB present in food could help avoiding problems of contamination into the food chain.

## Author Contributions

All authors listed, have made substantial, direct and intellectual contribution to the work, and approved it for publication.

## Conflict of Interest Statement

The authors declare that the research was conducted in the absence of any commercial or financial relationships that could be construed as a potential conflict of interest.
